# Does tidal volume challenge improve the feasibility of pulse pressure variation in patients mechanically ventilated at low tidal volumes? A systematic review and meta-analysis

**DOI:** 10.1186/s13054-023-04336-6

**Published:** 2023-02-02

**Authors:** Xiaoying Wang, Shuai Liu, Ju Gao, Yang Zhang, Tianfeng Huang

**Affiliations:** 1 Department of Anesthesiology, Northern Jiangsu People’s Hospital Affiliated to Yangzhou University, No.98, Nantong Xi Road, Guanglin District, Yangzhou, 225001 China; 2 Department of Burns and Plastic Surgery, The Affiliated Hospital of Yangzhou University, Yangzhou University, Yangzhou, Jiangsu China

**Keywords:** Tidal volume challenge, Pulse pressure variation, Change, Low tidal volume, Fluid responsiveness

## Abstract

**Background:**

Pulse pressure variation (PPV) has been widely used in hemodynamic assessment. Nevertheless, PPV is limited in low tidal volume ventilation. We conducted this systematic review and meta-analysis to evaluate whether the tidal volume challenge (TVC) could improve the feasibility of PPV in patients ventilated at low tidal volumes.

**Methods:**

PubMed, Embase and Cochrane Library inception to October 2022 were screened for diagnostic researches relevant to the predictability of PPV change after TVC in low tidal volume ventilatory patients. Summary receiving operating characteristic curve (SROC), pooled sensitivity and specificity were calculated. Subgroup analyses were conducted for possible influential factors of TVC.

**Results:**

Ten studies with a total of 429 patients and 457 measurements were included for analysis. The predictive performance of PPV was significantly lower than PPV change after TVC in low tidal volume, with mean area under the receiving operating characteristic curve (AUROC) of 0.69 ± 0.13 versus 0.89 ± 0.10. The SROC of PPV change yielded an area under the curve of 0.96 (95% CI 0.94, 0.97), with overall pooled sensitivity and specificity of 0.92 (95% CI 0.83, 0.96) and 0.88 (95% CI 0.76, 0.94). Mean and median cutoff value of the absolute change of PPV (△PPV) were 2.4% and 2%, and that of the percentage change of PPV (△PPV%) were 25% and 22.5%. SROC of PPV change in ICU group, supine or semi-recumbent position group, lung compliance less than 30 cm H_2_O group, moderate positive end-expiratory pressure (PEEP) group and measurements devices without transpulmonary thermodilution group yielded 0.95 (95%0.93, 0.97), 0.95 (95% CI 0.92, 0.96), 0.96 (95% CI 0.94, 0.97), 0.95 (95% CI 0.93, 0.97) and 0.94 (95% CI 0.92, 0.96) separately. The lowest AUROCs of PPV change were 0.59 (95% CI 0.31, 0.88) in prone position and 0.73 (95% CI 0.60, 0.84) in patients with spontaneous breathing activity.

**Conclusions:**

TVC is capable to help PPV overcome limitations in low tidal volume ventilation, wherever in ICU or surgery. The accuracy of TVC is not influenced by reduced lung compliance, moderate PEEP and measurement tools, but TVC should be cautious applied in prone position and patients with spontaneous breathing activity.

*Trial registration* PROSPERO (CRD42022368496). Registered on 30 October 2022.

**Supplementary Information:**

The online version contains supplementary material available at 10.1186/s13054-023-04336-6.

## Introduction

Fluid administration remains the first-line therapy wherever in ICU or operation room (OR). Both fluid overload and insufficient could cause deleterious effects, such as pulmonary edema, tissue hypoperfusion [1, 2]. However, only half of the patients are fluid responsiveness in clinical work [3].

Dynamic indices derived from arterial wave change, based on heart–lung interaction during mechanical ventilation, such as pulse pressure variation (PPV) or stroke volume variation (SVV), are proven to be superior than static indices [4, 5]. Among these indices, PPV is more reliable, quickly accessible from bedside and more extensively studied, and has been widely used in critically ill patients [4, 6]. However, since mechanical ventilation can trigger cardiac preload change in periodicity, in the condition of no arrhythmias and closed thoracic cavity, low tidal volumes are insufficient to produce significant change in thoracic pressure, so is the preload. This would add false negative results in fluid responsive patients, making PPV and other dynamic hemodynamic indices inaccurate [7–9]. It has been reported that PPV is reliable when tidal volume at least 8 ml/kg predicted body weight (PBW) [7]. But nowadays, low tidal volume ventilatory strategy (usually 6 ml/kg), improving outcomes and reducing pulmonary complications, has been widely used in ICU or general anesthesia surgery patients [10–12], which further restricts the application of PPV.

The concept of tidal volume challenge (TVC) was proposed to solve the dilemma for PPV in a concise manner [13]. The procedure of TVC is to adjust volume tidal from 6 (PBW) to 8 ml/kg (PBW) and obtains the increasing preload dependence of right ventricle and decreases the venous return, which cause patients more fluid responsive [13]. Many recent studies reported the change of PPV after TVC, including the absolute or percentage change of PPV (△PPV or △PPV%), in the assessment of fluid responsiveness in low tidal volume ventilation patients [14–23]. However, the results were conflicting and a recent meta-analysis of TVC only included 3 original studies in the early years [8]. Besides, except low tidal volume, some factors that would possibly influence intrathoracic pressure were existed in these study settings, such as position [24], lung compliance [25] or positive end-expiratory pressure (PEEP) [26].

We conducted this systematic review and meta-analysis to assess the ability of TVC to help PPV overcome the limitation in low tidal volume ventilation patients, and explore whether the factors influencing intra-thoracic pressure or other factors could possibly influence the accuracy of TVC.

## Material and methods

This meta-analysis was conducted according to the Preferred Reporting Items for Systematic Reviews and Meta-Analyses guidelines [27].

### Registration and protocol

This meta-analysis was registered on PROSPERO (CRD42022368496).

### Search strategy

Two authors independently searched relevant studies up to October 2022 in PubMed, Embase and Cochrane Library with the following terms and their combination: “tidal volume challenge” AND “pulse pressure variation” AND (“low tidal” OR” low tidal ventilation” OR “protective ventilation”) AND (“fluid responsiveness” OR “volume responsiveness”). All scanned abstracts, studies and citations were reviewed. If discrepancy existed, it was solved by the third arbitration. Moreover, references of the retrieved manuscripts were also manually cross-searched for further relevant publications.

### Selection criteria

The inclusion criteria were as follows (according to PICO):

S (study design): diagnostic experiments of TVC in fluid responsive assessment in low tidal volume ventilation patients.

P (patients): adult patients under low tidal volume ventilation in ICU or OR.

**Fig. 1:**
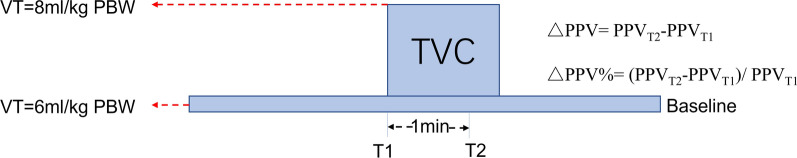
Concept of TVC and PPV change. *TVC* tidal volume challenge, *△*PPV absolute change of pulse pressure variation, *△PPV%* percentage change of pulse pressure variation, *Vt* tidal volume, *T1* measurement time point before TVC, *T2* measurement time point after the TVC start, *PBW* predicted body weight

I (interventions):change of PPV after TVC; TVC in the studies was defined as adjusting tidal volume from 6 to 8 ml/kg, and the measurements are performed one minute after TVC; △PPV defined as PPVvt8-PPVvt6 and △PPV% defined as (PPVvt8-PPVvt6)/ PPVvt6. The concepts of TVC and PPV change are presented in Fig. 1.

C (controls): fluid responsiveness assessment was performed with fluid challenge or response to PLR or its surrogates.

O (outcomes): the ability of TVC to improve the feasibility of PPV in low tidal volume ventilation.

(6) Others: studies published with full-text in any language; studies providing sufficient data for constructing 2-by-2 tables, including true positive (TP), false positive (FP), true negative (TN) and false negative (FN) [28].

We excluded those studies as follows: (1) studies with patients under normal or high tidal volume ventilation; (2) studies that used the same population or overlapping database; (3) studies without mechanically ventilation or spontaneously breathing patients; (4) animal studies; (5) studies on ventilated children.

### Date extraction and quality assessment

Two authors independently browsed the research indicators of the included studies. Extracted data included three parts: (1) basic information about the research such as the number of patients, study year and places, study indicators, measurement tools and ventilation settings; (2) the statistical results, including sensitivity, specificity, AUROC of the change of PPV and cutoff value; (3) discrepancies among studies that could be the heterogeneity or potential factors that could influence the degree of preload change caused by TVC, such as patients, position, lung compliance, ventilator settings, measurement tools.

Two investigators independently assessed the included studies by Diagnostic Accuracy Studies-2 (QUADAS-2) recommended by the Cochrane Handbook [29]. The QUADAS-2 tool consists of four domains: patient selection, index test, reference standard and flow and timing. All domains were evaluated in terms of risk of bias and would be answered as “yes,” “no” and “unclear.” “Unclear” was defined if the original study failed to provide adequate information that the authors had difficulty to judge. The risk could be defined as low under the circumstance of a consistency of “yes.” Quality assessment was performed by RevMan software 5.3.

### Statistical analysis

The bivariate mixed-effects regression model was performed in data synthesis to incorporate the negative correlation, which might arise between the sensitivity and specificity [30, 31]. We estimated overall pooling of sensitivity, specificity and diagnostic odds ratio (DOR) with 95% confidence interval (CI) using a bivariate random-effects model. Summary receiver operating characteristic curve (SROC) was potted and the area under the summary receiver operating characteristic curve (AUSROC) was calculated by Rutter and Gatsonis test [32]. Operative performance quality was graduated according to Fisher et al [33]. Diagnostic power was outstanding if the AUROC was more than 0.9 and was poor if the AUROC was less than 0.7 [34].The cutoff values of PPV change were performed in the scatter plot to observe the distribution, dispersion, central tendency and extremum.

Heterogeneity between studies was quantitatively assessed by the Chi-square test and Cochran’s Q test. *P* value for Q test < 0.1 or *I*^2^ > 50% was considered existing significant heterogeneity. Heterogeneity caused by the threshold effect in the diagnostic test was calculated in the Spearman correlation coefficient, which was estimated by the Moses–Shapiro–Littenberg [35]. If correlation coefficient was 1, which means the proportion of heterogeneity likely due to threshold effect was 100%, meta-regression was unnecessary [36]. Other methods, such as rule out one single study one by one to find heterogeneity sources, were also conducted.

Subgroup analysis was conducted according to factors which could possibly affecting intrathoracic pressure and the predictability of TVC. Since patients in ICU are more complicated, and the results of TVC in ICU were contradictory, we conducted ICU groups. We also conducted subgroup analysis including supine or semi-recumbent group, lung compliance < 30 cm H_2_O group, moderate PEEP group (5 cm H_2_O ≤ PEEP ≤ 15 cm H_2_O) and measurement tools without TPTD group. Spontaneous breathing subgroup was also observed. Since some studies included both △PPV and △PPV%, the primary and more accurate indicator was calculated when in analysis. Diagnostic accuracy parameters between groups were compared using the likelihood ratio Chi-square test if necessary.

Public bias was estimated by Deek’s funnel plot asymmetry test, with *P* < 0.1 indicating statistical significance [37].

The description of data was expressed as mean (95% CI) or as mean ± standard deviation. Meta-analysis was performed by Stata 15. 0 (StataCorp, College Station, TX) with the Midas module. A two-tailed *P* < 0.05 was considered statistically significant.

## Results

### Characteristics of included studies

**Fig. 2:**
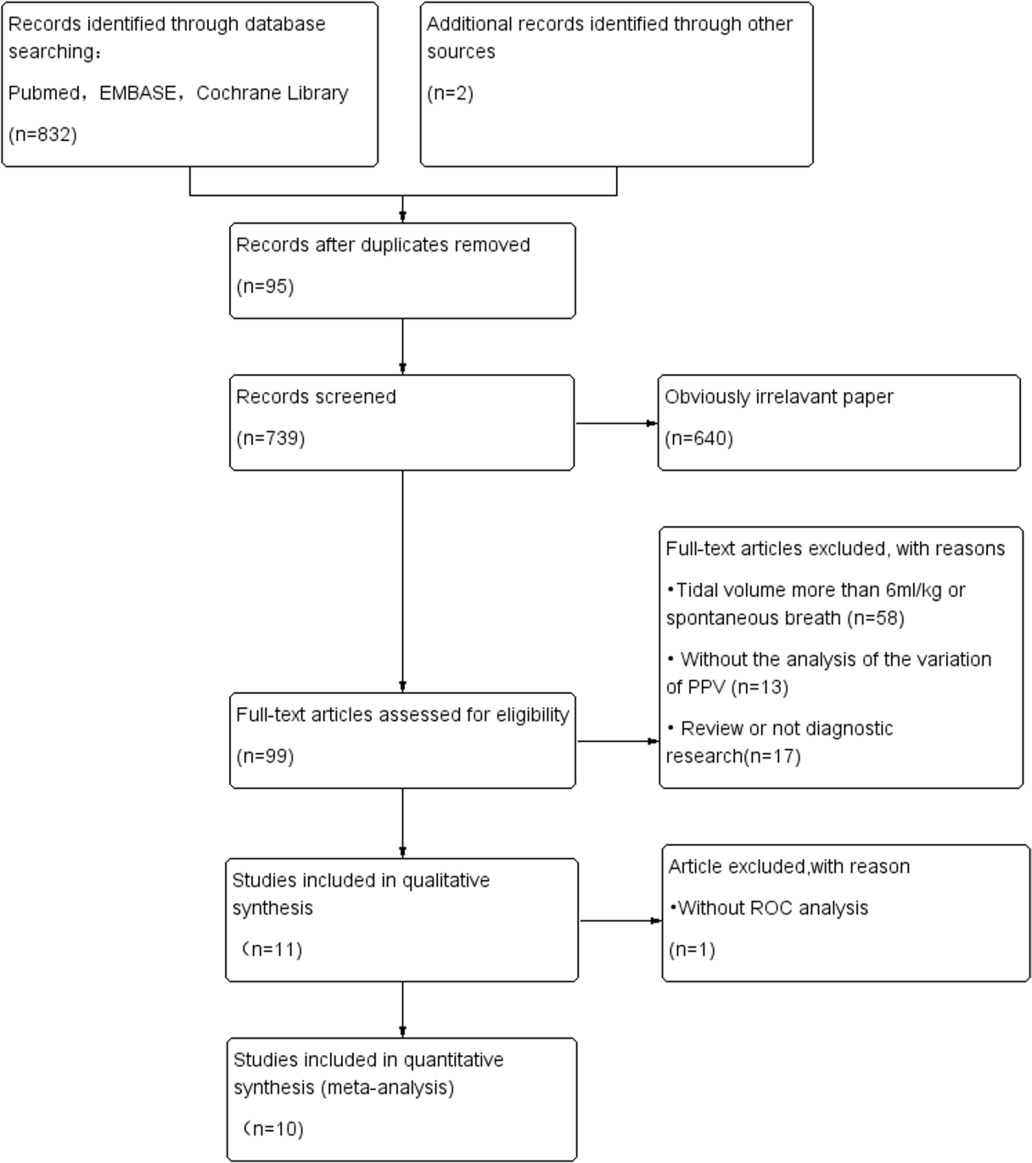
Flow of studies selection

**Fig. 3:**
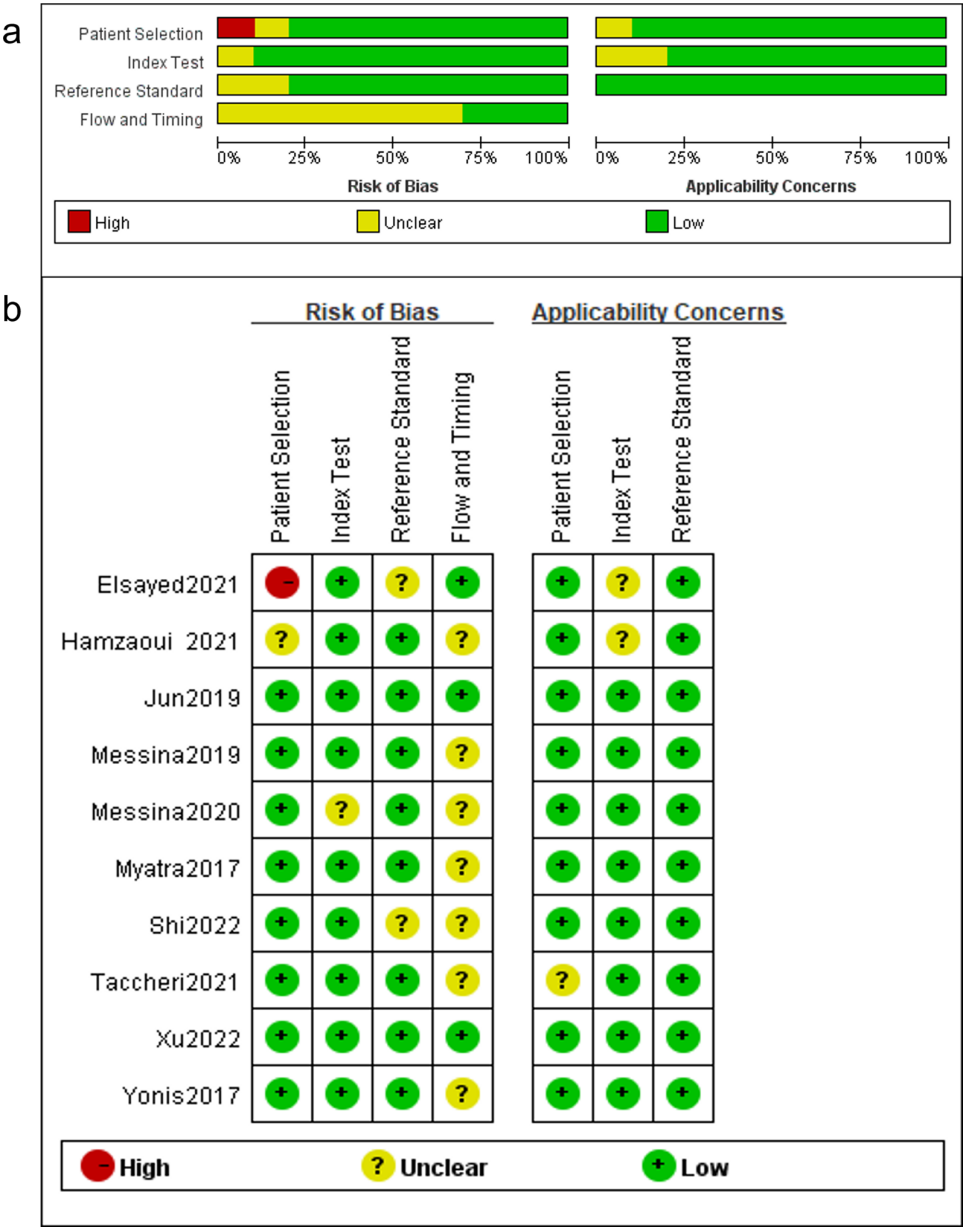
Risk of bias and applicability concerns for the studies included in the meta-analysis. **a** Risk of bias graph. **b** Risk of bias summary

Our meta-analysis yielded 834 primary studies after initially screened; 735 studies were excluded because of obviously irrelevant and duplicates. In the remaining 99 studies, full-text manuscripts were screened and 88 full-text articles were excluded because of tidal volume more than 6 ml/kg or spontaneous breath, without the analysis of the variation in PPV after TVC, and review or not diagnostic research. Further screening the remaining 11 full articles, one was excluded because of ROC analysis missing. Finally, 10 studies with a total of 429 patients and 457 measurements were included in the meta-analysis, presented in Fig. 2. QUADAS-2 was presented in Fig. 3.

Characteristics of the included studied were summarized in Table 1. A total of 215 measurements (47.7%) were fluid responsive in the overall studies. 7 studies were conducted in ICU, while the left in OR. The position was varied, 6 studies were in supine or semi-recumbent position, while 3 studies were in prone position and 1 in Trendelenburg position. The mean lung compliance ranged from 16.8 to 83 ml/cm H_2_O. Except one study missing data, the mean PEEP ranged from 5 to 14 cm H_2_O. In the overall 10 studies, 5 studies acquired PPV with transpulmonary thermodilution (TPTD) and 5 studies with others.

**Table 1:** General characteristics of selected studies

Study/year	Patients	Setting	Sample size	Position during study	Primary /study predictor of PPV change	Secondary predictor of PPV change	PPV acquirement	Reference preload change	Reference standard	Reference standard acquirement	Tidal volume (ml/kg)	Mean lung compliance (ml/cm H_2_O)	Mean PEEP (cmH_2_O)	SB activity
Myatra2017 [14]	Acute circulatory failure patients	ICU	30	Supine	△PPV	△PPV%	PiCCO	VE 6 ml/kg PBW	CI ≥ 15%	TPTD	6	28.0 ± 7.0	8.4 ± 2.9	Avoid
Yonis 2017 [15]	ARDS patients	ICU	19	Prone	△PPV%	N	PiCCO	VE 500 ml crystalloids	CI ≥ 15%	TPTD	6	30 (23, 39)	8 (5, 10)	Avoid
Jun2019 [16]	Patients undergoing robot-assisted laparoscopic surgery	OR	38	Trendelenburg	△PPV	△PPV%	CARESCAPE monitor	VE6ml/kg colloid	SVI ≥ 15%	ODM	6	16.8	5	Avoid
Messina2019 [17]	Patients undergoing neurosurgery	OR	40	Supine	△PPV%	N	MostCare	VE 250 ml crystalloids	SVI ≥ 10%	MostCare	6	65 (58, 73)	5 (5, 5.5)	Avoid
Messina2020 [18]	Prone Neurosurgical Patients	OR	40	Prone	△PPV%	N	MostCare	VE 250 ml crystalloids	SVI ≥ 10%	MostCare	6	65	5	Avoid
Elsayed2021 [19]	Hemodynamic instability patients	ICU	46	Semi-recumbent	△PPV	N	Mindray monitor	VE 4 ml/kg	CI ≥ 15%	ODM	6	28.0 ± 6.0	unclear	Avoid
Taccheri2021 [20]	Mechanical ventilated patients	ICU	30	Semi-recumbent	△PPV	△PPV%	PiCCO	PLR	CI ≥ 10%	TPTD	6	31.5 ± 12.3	10.5 ± 3.3	Avoid
Hamzaoui2021 [21]	critically ill adult patients	ICU	54	semi-recumbent	△PPV	N	Hemodynamic monitors	PLR	VTI ≥ 12%	TTE	6.0 (5.9, 6.3)	41 (36, 46)	10 (9, 11)	Yes
Shi2022 [22]	ARDS patients	ICU	84	Prone	△PPV	N	PiCCO	Both Trendelenburg EEO8	CI ≥ 8% CI ≥ 5%	TPTD	6	32 (22, 41)	14 (11, 16)	Avoid
Xu2022 [23]	Septic shock patients	ICU	76	Supine	△PPV	N	PiCCO	VE 250 ml crystalloids	CI ≥ 15%	TPTD	6	25.7 ± 3.8	9.5 ± 2.8	Avoid

*PiCCO* pulse contour cardiac output, *TPTD* transpulmonary thermodilution, *ODM* esophageal Doppler monitor, *iMEC10* Mindray monitor, *OR* operation room, *ICU* intensive care unit, *RDS* acute respiratory distress syndrome, *△PPV* absolute change of pulse pressure variation, *△PPV%* percentage change of pulse pressure variation, *EEO*_*8*_ end-expiratory occlusion performed at 8 ml/kg tidal volume, *VE* volume expansion, *CI* cardiac index, *SVI* stroke volume index, *TTE* Trans-thoracic echocardiography, *VTI* velocity–time integral, *AUROC* area under the receiver operator characteristics curve, *N* none, *PEEP* positive end-expiratory pressure, *SB* spontaneous breathing. Data were presented with 95% confidence interval or mean ± standard deviation if possible

### Performance of TVC improve the accuracy of PPV in low tidal volume ventilation

**Fig. 4:**
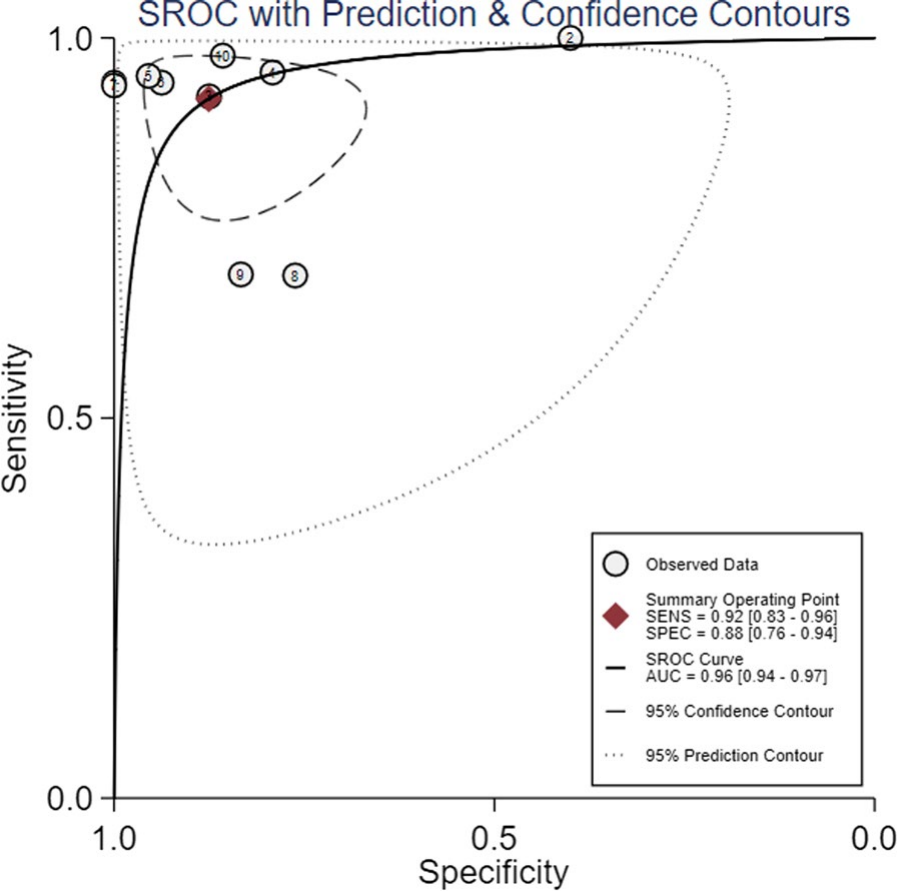
Summary receiver operating characteristic curve for the change of pulse pressure variation after tidal volume challenge predicting fluid responsiveness in low tidal volume ventilation. The diamond is the summary point representing the average sensitivity and specificity estimates. *AUC* area under the curve, *SENS* sensitivity, *SPEC* specificity, *SROC* summary receiver operating characteristics. The ellipses around this summary point are the 95% confidence region (dashed line) and the 95% prediction region (dotted line)

**Fig. 5:**
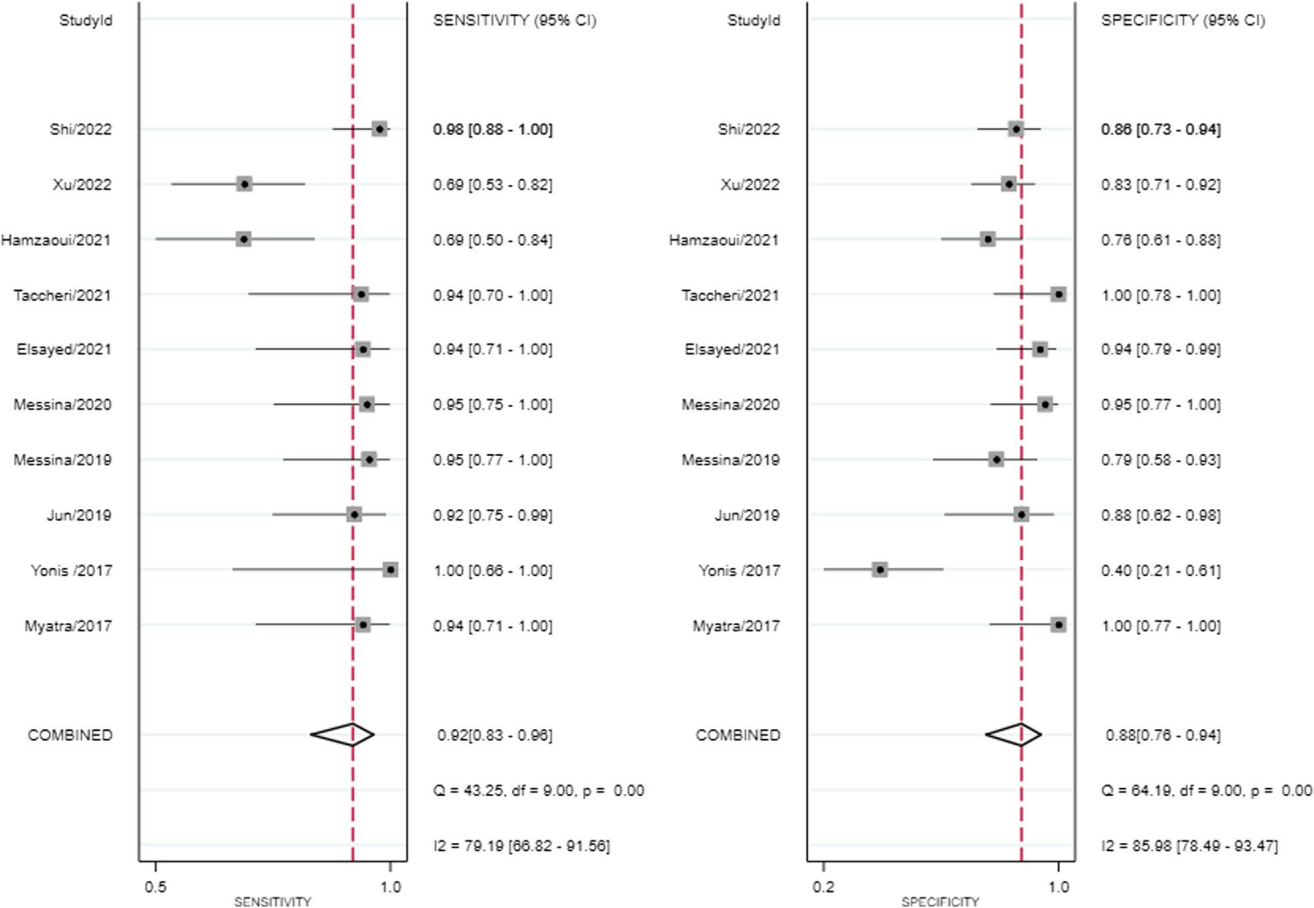
Sensitivity and specificity of the change of pulse pressure variation after tidal volume challenge predicting fluid responsiveness in low tidal volume ventilation for all data. Each solid square represents an individual study. Error bars represent 95% CI

The predictive performance of PPV was significantly lower than PPV change in low tidal volume, with mean AUROC of 0.69 ± 0.13 versus 0.89 ± 0.10, *P* < 0.01, presented in Table 2. SROC of the PPV change yielded an area under the curve of 0.96 (95% CI 0.94, 0.97) with overall *I*^2^ of 76% (95%47, 100), presented in Fig. 4 The pooled sensitivity and specificity of the change of PPV were 0.92 (95% CI 0.83, 0.96) and 0.88 (95% CI 0.76, 0.94) with *I*^2^ of 79% (95%67, 92) and 86% (95%78, 93) separately, presented in Fig. 5. DOR of the change of PPV was 81 (95% CI 23, 284) with *I*^2^ of 67% (95% CI 0, 86).

**Table 2:** Predictive performance of PPV change after TVC in low tidal mechanically ventilated patients

Study/year	predictor	Subjective numbers could be calculated				Threshold (%)	Sensitivity	Specificity	AUROC of PPV change	AUROC of PPV
		TP	FP	FN	TN					
Myatra2017 [14]*	△PPV	16	0	1	14	3.5	0.94	1.00	0.99 (0.98, 1.00)	0.69
	△PPV%	16	0	1	14	48	0.94	1.00	0.97 (0.92, 1.00)	
Yonis 2017 [15]	△PPV%	9	15	0	10	29	1.00 (0.66, 1.00)	0.40 (0.1, 0.7)	0.59 (0.31, 0.88)	0.49 (0.21, 0.77)
Jun2019 [16]*	△PPV	24	2	2	14	1	0.92 (0.73, 0.99)	0.86 (0.57, 0.98)	0.95 (0.83, 0.99)	0.69 (0.52, 0.83)
	△PPV%	24	4	5	14	25	0.83 ((0.63, 0.95)	0.79 (0.49, 0.95)	0.87 (0.72, 0.96)	
Messina2019 [17]	△PPV%	21	5	1	19	13.3	0.95 (0.74, 1.00)	0.76 (0.53, 0.92)	0.94 (0.82, 0.99)	0.68 (0.50, 0.85)
Messina2020 [18]	△PPV%	19	1	1	21	12.2	0.95	0.95	0.96 (0.87, 1.00)	0.69
Elsayed2021 [19]	△PPV	16	2	1	30	3.5	0.94	0.94	0.96	0.85
Taccheri2021 [20]*	△PPV	15	0	1	15	1	0.93 (0.68, 1.00)	1.00 (0.78, 1.00)	0.98 ± 0.02	0.66
	△PPV%	15	2	1	15	20	0.93 (0.68, 1)	0.87 (0.59, 0.98)	0.94 ± 0.04	
Hamzaoui2021 [21]	△PPV	22	10	10	32	2	0.69	0.76	0.73 (0.60, 0.84)	0.61 (0.48, 0.75)
Shi2022 [22]	△PPV	42	7	1	42	3.5	0.98 (0.89, 0.99)	0.86 (0.75, 0.79)	0.94 (0.88, 0.99)	0.85 (0.77, 0.92)
Xu2022 [23]	△PPV	31	9	14	45	2	0.84	0.84	0.90 (0.81, 0.96)	0.69 (0.57, 0.79)

*PPV* pulse pressure variation, *△PPV* absolute change of pulse pressure variation, *△PPV%* percentage change of pulse pressure variation, *Sen* sensitivity, *Spec* specificity, *AUROC* area under the receiver operator characteristics curve, *TP* true positive, *FP* false positive, *FN* false negative, *TN* truth negative

*The studies including both △PPV and △PPV%. Data were presented with 95% confidence interval or mean ± standard deviation if possible

### Heterogeneity investigation

Significant heterogeneity in the 10 studies was observed with an overall *Q* = 8.3, *I*^2^ = 76%, *P* < 0.01. The Spearman correlation coefficient was 0.09; however, significant heterogeneity was not found in the latter meta-regression analysis (presented in Additional file 1: Fig. S1). We attempted to remove the study of Yonis et al. [15] in the analysis. The overall heterogeneity was significant decreased to *I*^2^ = 42% and *Q* = 3.605, *P* = 0.09. Spearman correlation coefficient was 1, whereas the value of *I*^2^ and Q test changed insignificantly when we removed any other study in the analysis (presented in Additional file 2: Table S1 and Additional files 3, 4: Figs. S2, S3).

### Subgroup analysis

In ICU groups, TVC has good predictability in ICU with SROC yielding 0.95 (95%0.93, 0.97), pooled sensitivity of 0.91 (95%0.77, 0.97) and pooled specificity of 0.88 (95%0.69, 0.96), presented in Additional file 5: Fig. S4. The change of PPV after TVC in supine or semi-recumbent group, lung compliance < 30 cm H_2_O group, moderate PEEP group and measurement tools without TPTD group all performed good prediction of fluid responsiveness with SROC yielded the area of 0.95 (95% CI 0.92, 0.96), 0.96 (95% CI 0.94, 0.97), 0.95 (95% CI 0.93, 0.97) and 0.94 (95% CI 0.92, 0.96) separately (presented in Additional file 6: Fig. S5 and Table 3). However, the lowest AUROC of PPV change was 0.59 (95% CI 0.31–0.88) in prone position and 0.73 (95% CI 0.60–0.84) in patients with spontaneous breathing activity.

**Table 3:** Subgroup analysis and heterogeneity source

Subgroups	Samples	AUROC (95% CI)	Sensitivity (95% CI)	Specificity (95% CI)	DOR (95% CI)	*I*^2^ (%) (95% CI)	Q	*P* value	Spearman correlation coefficient	Statistical heterogeneity	Heterogeneity source
Patients in ICU group	7	0.95 (0.93, 0.97)	0.91 (0.77, 0.97)	0.88 (0.69, 0.96)	72 (13, 396)	77 (74, 100)	8.53	< 0.01	0.22	Significant	Others
Supine or semi-recumbent	6	0.95 (0.92, 0.96)	0.88 (0.73, 0.95)	0.89 (0.79, 0.95)	62 (13, 297)	0 (0, 100)	0.42	0.41	1	Very low	Totally threshold effect
Low lung compliance < 30 cm H_2_O	4	0.96 (0.94, 0.97)	0.89 (0.72, 0.96)	0.91 (0.81, 0.96)	87 (15, 506)	0 (0, 100)	0.51	0.39	1	Very low	Totally threshold effect
PEEP ≥ 5 cm H_2_O and ≤ 15 cm H_2_O group	9	0.95 (0.93—0.97)	0.92 (0.82, 0.97)	0.86 (0.73, 0.94)	72 (19, 270)	77 (51, 100)	8.87	< 0.01	0.19	Significant	Others
Measure tools except TPTD	5	0.94 (0.92, 0.96)	0.91 (0.78, 0.97)	0.87 (0.78, 0.93)	70 (16, 308)	0 (0, 100)	0.86	0.33	1	Very low	Totally threshold effect
Overall data except Yonis 2017	9	0.94 (0.92, 0.96)	0.92 (0.82,0.96)	0.88 (0.82,0 .92)	83 (26, 260)	42 (0, 100)	3.5	0.09	1	Low	Totally threshold effect
Overall date	10	0.96 (0.94, 0.97)	0.92 (0.83, 0.96)	0.88 (0.76, 0.94)	81 (23, 284)	76 (47, 100)	8.3,	< 0.01	0.06	Significant	Others

*TPTD* transpulmonary thermodilution, *PEEP* positive end-expiratory pressure, *AUROC* area under the receiver operator characteristics curve, *DOR* diagnostic: odds ratio, *CI* confidence interval, *Q* Cochran’s Q test, *I*^2^ inconsistence*y*

### The comparison of △PPV and △PPV%

**Fig. 6:**
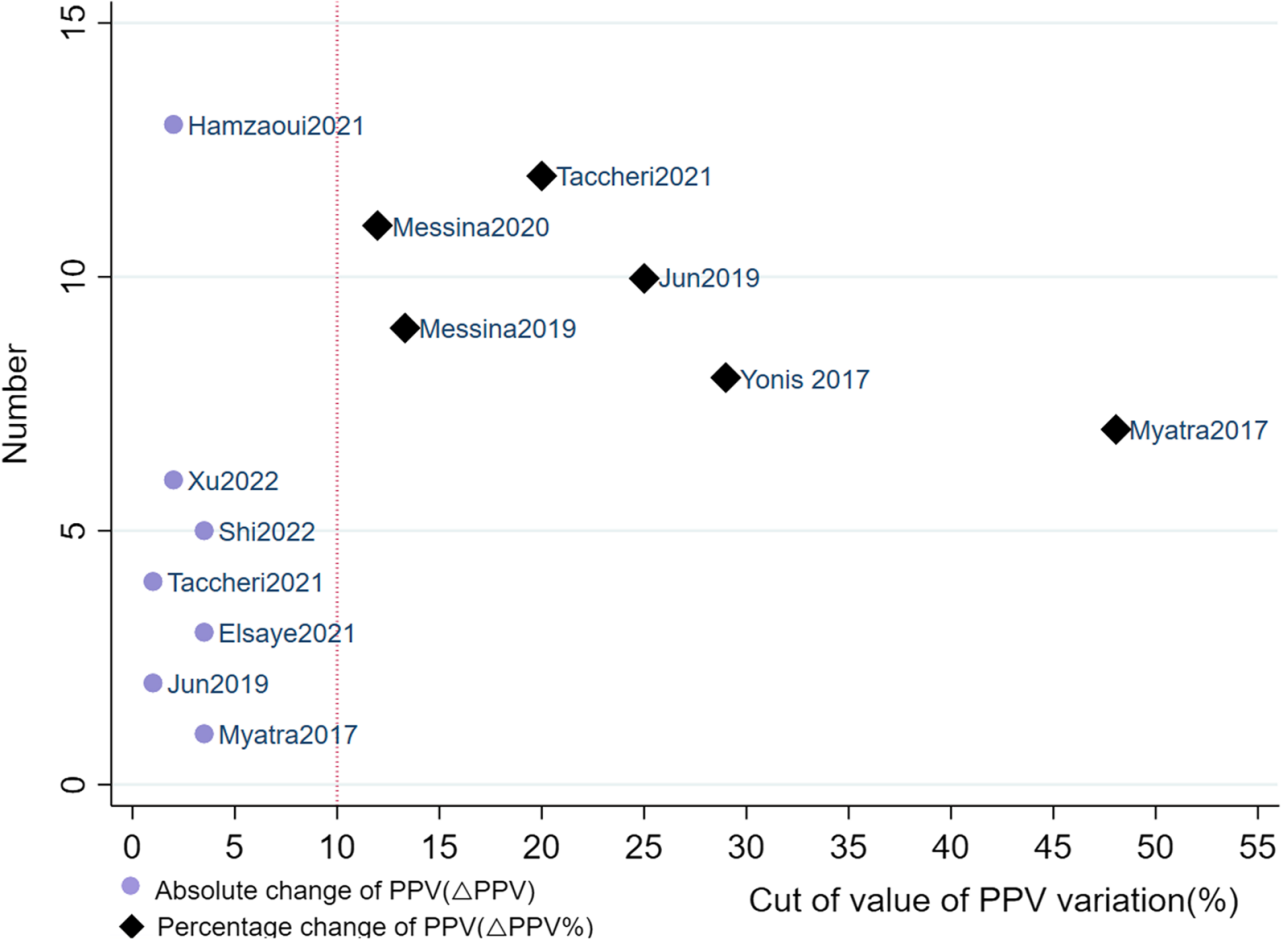
Scatter plot of cutoff value of △PPV and △PPV% in included studies. The purple black dots represent absolute change of pulse pressure variation (△PPV). The black diamond squares represent percentage change of pulse pressure variation (△PPV%). The cutoff values of △PPV are as follows: (1) Myatra 2017 [14], 3.5%; (2) Jun 2019 [16], 1%; (3) Elsayed 2021 [19], 3.5%; (4) Taccheri 2021 [20], 1%; Hamzaoui2021 [21]; (5) Shi 2022 [22], 3.5%; (6) Xu 2022 [23], 2%. The cutoff values of △PPV% are as follows: (1) Myatra 2017 [14], 48%; (2)Yonis 2017 [15], 29%; (3) Jun 2019 [16], 25%; (4) Messina 2019 [17], 13.3%; (5) Messina 2020 [18], 12%; (6) Taccheri 2021 [20], 20%;

In groups comparison, there is no difference in AUC > 0.9 rate, △PPV versus △PPV%, *p* = 0.31. But interestingly, in the same study, △PPV always perform slightly better than △PPV%(presented in Table 2). The SROC of △PPV and △PPV% yielded the area of 0.94 (95% CI 0.92, 0.96) and 0.96 (95% CI 0.94, 0.97), with *I*^2^ of 19% (95% CI 0, 100) and 78% (95% CI 52, 100), presented in Additional file 7: Fig. S6. The mean and median cutoff values of △PPV were 2.4% and 2%, ranged from 1 to 3.5%, and that of △PPV% were 25% and 22.5%, ranged from 12 to 48%, presented in Fig. 6.

### Public bias

**Fig. 7:**
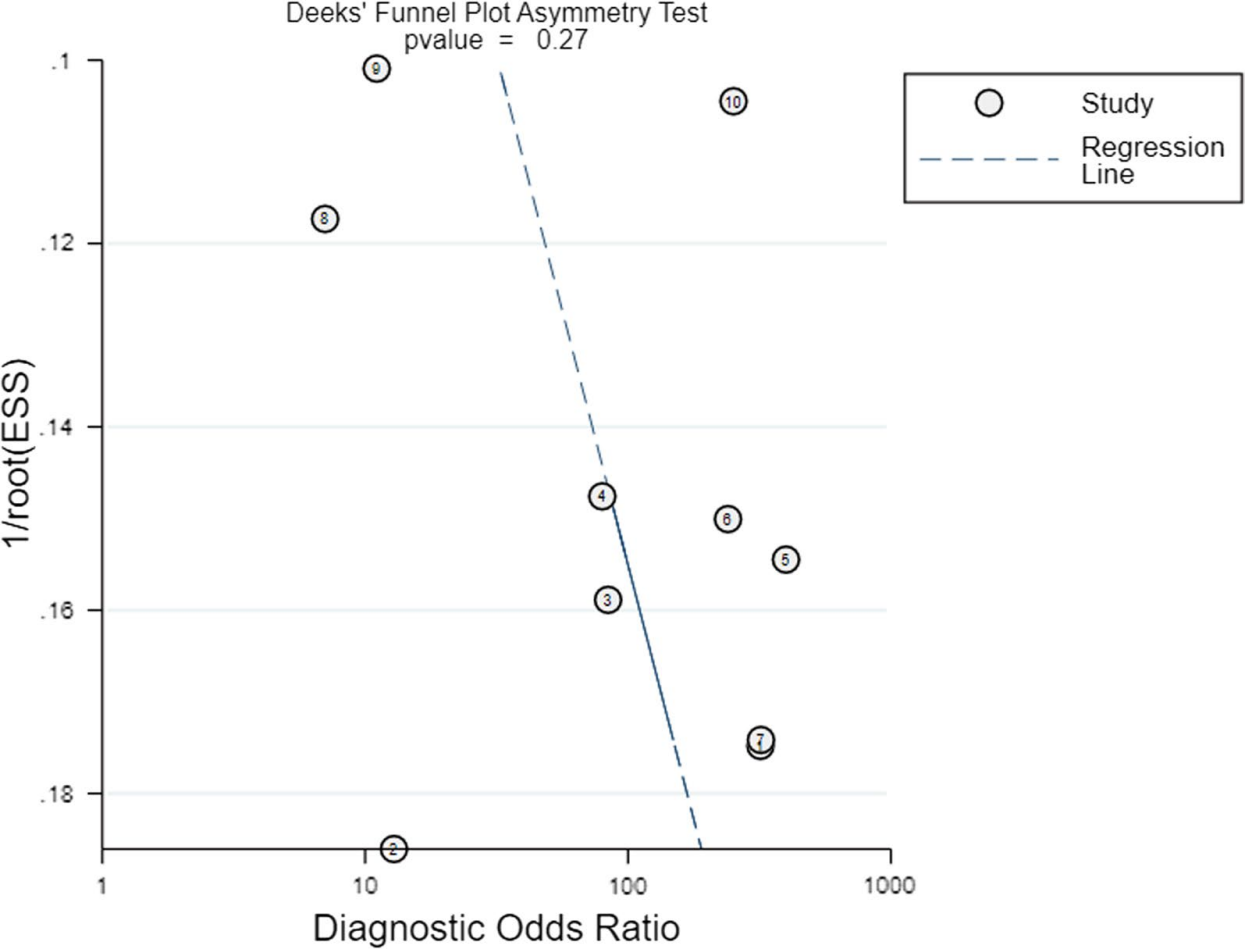
Deeks’ funnel plot with superimposed regression line. *P* value for slope coefficient is 0.27, which is greater than 0.05, suggesting the symmetry of the studies and the low likelihood of publication bias

The Deek’s funnel plot asymmetry test of the meta-analysis is shown in Fig. 7, and no significant public bias was found in our meta-analysis (*P* = 0.27).

## Discussion

**Fig. 8:**
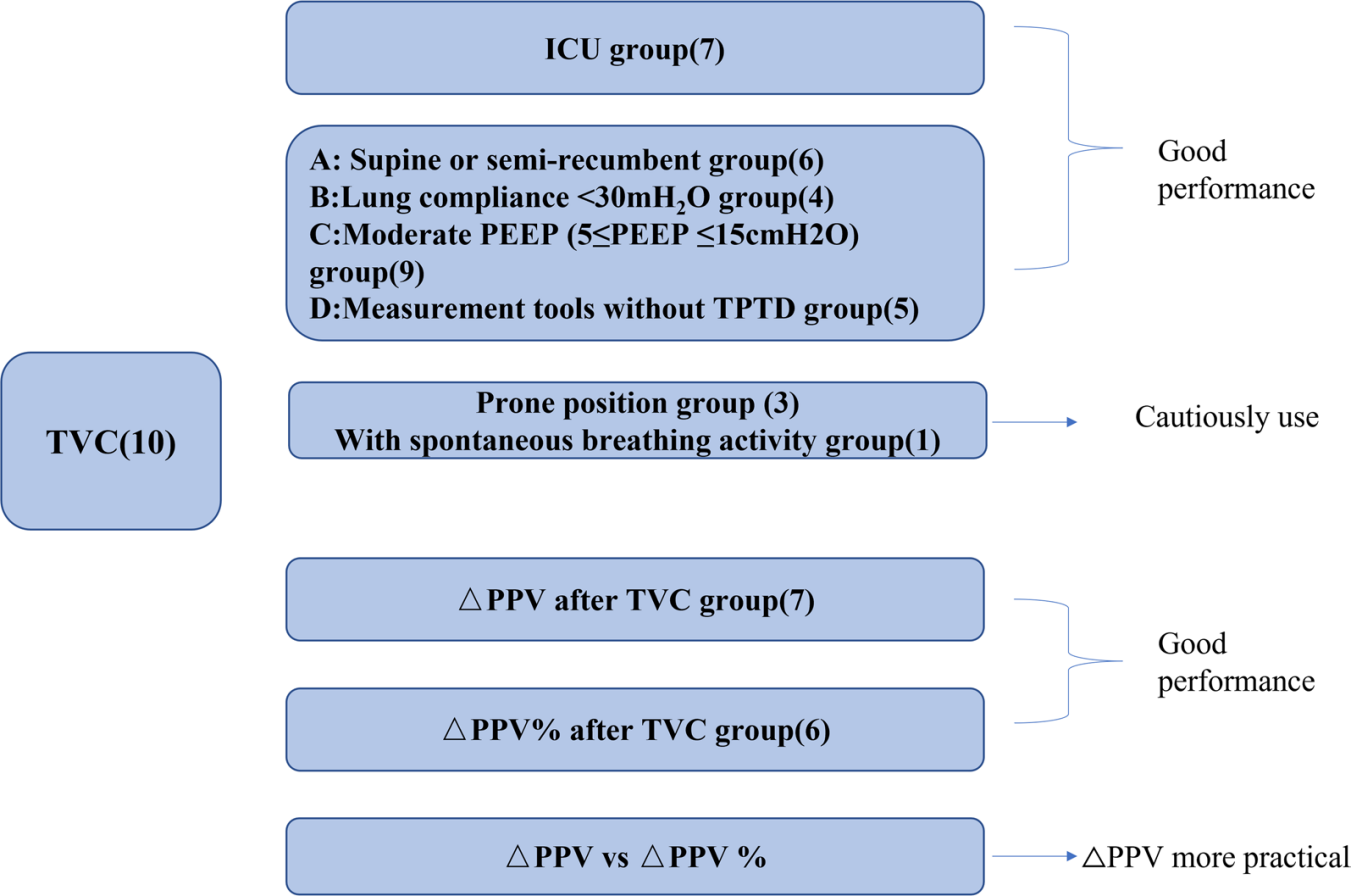
Study research string. *TVC* tidal volume challenge, *△PPV* absolute change of pulse pressure variation, *△PPV%* percentage change of pulse pressure variation, *TPTD* transpulmonary thermodilution, *PEEP* positive end-expiratory pressure

This systematic review and meta-analysis mainly revealed that: (1) The change of PPV that caused by TVC is a good fluid responsiveness predictor in low tidal volume ventilation; (2) TVC is reliable in both ICU and OR, and the accuracy would not be affected by low lung compliance, moderate PEEP and the measurement devices of PPV; (3) But, TVC should be cautious applied in prone position and patients with spontaneous breathing activity. The exact research string of the whole study is presented in Fig. 8.

A recent meta-analysis showed PPV performed moderately in tidal volume less than 8 ml/kg due to not enough preload change triggered by mechanical ventilation [38]. Previous research observed PPV obviously increased 4.8% meanly in patients even 5 min after adjusting tidal volume from 6 to 8 ml/kg [39]; this challenge could help augmenting preload change when evaluating fluid responsiveness; soon after that, TVC was proposed [13]. As expected, in our meta-analysis, PPV change after TVC performed significantly better than PPV in low tidal volume ventilation, with SROC more than 0.9.

In subgroup analysis, patients in ICU are more complicated with shock, organ failure or other critically ill state, and the results were contradictory. But in statistical analysis, TVC is still highly reliable in ICU patients, with similar SROC to the overall data. Theoretically, the reduced lung compliance could enhance the transmission of airway pressure to the pericardium and the vena cava, thus, weaken the effect of SV change caused by ventilation [40, 41]. PPV was reported low predictability in lung compliance less than 30 cm H_2_O [25]. Fortunately, we found PPV change after TVC was less affected by reduced lung compliance, this could be relevant to the preload change weakened by low lung compliance is limited, and that was supplemented by TVC. Moderate PEEP could supply pressure on the end expiratory, enlarge cyclic cardiac output change caused by ventilation [26] and thus strength the effect of TVC. As a result, we found TVC performed well in patients with moderate PEEP. Except from the widely used and standard method TPTD, pulse contour analysis technique or noninvasive monitor which also acquires PPV is more convenient and noninvasive but more susceptible of interferences [5]. However, we observed that TVC was not influenced by measurement tools.

We found TVC would limit in some circumstances. In a patient with spontaneous breathing activity, the TVC may fail because of asynchronism between the increased Vt and the breathing pattern of the patient. This may cause a contrast between the patient and ventilator, affecting the right ventricle afterload and, hence, the changes in right ventricle stroke volume. As expected, TVC performs obviously bad with AUROC of only 0.73 in patients with spontaneous breathing activity [21], which is much lower than that in totally mechanical ventilation studies. The results of TVC were also contradictory in prone position [15, 18, 22]. Physiologically, the venous return could be impeded when intra-abdominal pressure is more than right atrial pressure because the abdominal inferior vena cava collapses and a vascular waterfall develops at the level of the diaphragm [24]; this could cause TVC fail to decrease preload. Different clinical settings were also accountable, but we disagreed with Shi et al. [22] who accounted the contradictory results to lung compliance since we found TVC was unaffected by reduced lung compliance. Besides, recent meta-analysis of EEO, the similar theory of heart–lung interaction functional test to TVC, was also proved to be limited in prone position [42].

The cutoff values of PPV change were varied in our study. In fact, this is the common phenomenon caused by different preload state before TVC. The extreme cutoff value of △PPV% was 48% in Mytra’ study [14]; this could be related to the fact that the selected people were circulatory failure patients, who were sensitive to preload change. In the study of Jun [16], the included patients were normal hemodynamic state but with extremely reduced lung compliance of 16.8 cm H_2_O. However, low lung compliance induces insignificant preload change due to more obstruction stress from chess or pulmonary to cardiac or vena, and consequently, the final cutoff value of △PPV was only 1%. Besides, some ventilation settings that could increase cyclic changes of intrathoracic pressure, such as PEEP [26] and larger tidal volume [7], as a result, acquire larger cutoff value.

To a certain extent, △PPV% is a surrogate of △PPV and they possessed the same tendency of predictability, whereas we found △PPV% was less practical and reliable than △PPV, which was in the agreement with Myatra [14]. Initially, heterogeneity of △PPV% group was significant and larger than △PPV group. Secondly, the AUSROC of △PPV% performed a slighter lower than △PPV when in the same studies [14, 16, 20]. Moreover, the cutoff values of △PPV were more central between 1 and 3.5%, while that of △PPV% were more dispersed in the scatter plot, which could cause more threshold effect heterogeneity and difficult to assess fluid responsiveness accurately. Last but not the least, the calculation of △PPV% is more complicated and not suitable for beside or emergency.

Some limitations in our meta-analysis should be acknowledged. Firstly, ten number of diagnostic studies was included with significant heterogeneity in the overall analysis representing a limitation of this study. Although the study of Yonis et al. [15] could be the heterogeneity of this meta-analysis, other potential heterogeneity should be considered. Secondly, due to some included researches missing data of sensitivity and specificity of PPV, we failed to calculate the SROC of PPV in low tidal volume ventilation in comparison. Instead, we statistically compare the AUROCs of PPV and PPV change in low tidal volume ventilation patients with original data. Thirdly, we did not perform the comparison of the opposite subgroups, such as low versus high lung compliance and prone versus supine, because, on the one hand, our main intention is to observe whether TVC is still reliable in some circumstance, like low lung compliance, some position, moderate PEEP and irregular measurement tools, rather than comparing the differences between the opposite two groups. After all, these conditions exit commonly in patients need lung protective strategies and probably influence TVC. On the other hand, some opposite groups only contained 2 or 3, or even 1 study, and small number subgroup is less convincing for comparison and has larger risk of statistical error. Fourthly, some other valuable target subgroups that could influence TVC were failed to analyze because of current studies restriction. Higher level of PEEP results in greater cyclic changes in preload [26], making patients more fluid responsive [43], while PEEP of 15 to 20 cm H_2_O could decrease cardiac output [44]. However, we failed to analyze PEEP more than 15 cm H_2_O group because all included studies used moderate PEEP. Spontaneous breathing activity during mechanical ventilation is common in ICU and TVC could fail in this condition, but currently, only one study proved that, and we failed to make summary statistical analysis. Fifthly, not all included studies used fluid challenge as golden standard preload challenge; this could bring more interferences to assess TVC. Actually, the essence of fluid responsiveness assessment is detecting preload change, apart from classic fluid challenge; other surrogates or called functional tests, such as PLR, mini-fluid challenge, EEO or Trendelenburg, could also trigger the same effect, and some even perform advantages over classic method [45].Thus, these studies with surrogates of fluid challenge are also vital and valuable. Finally, the current studies in our meta-analysis all used standard TVC (6 to 8 ml/kg Vt, 1 min). As we all know, TVC is the supplement of preload change; hence, change size of Vt or performance time could influence the SV change caused by TVC. Similar study of fluid challenge reported 100 ml and 250 ml crystalloid had the same effect in preload [46], but that decreased when performed over 30 min [47]. Overall, more researches are warrant in the future about TVC.

## Conclusion

TVC could improve the feasibility of PPV in patients mechanically ventilated at low tidal volumes by calculating PPV change after TVC. Both △PPV and △PPV % have good predictability, but △PPV is recommended first. TVC performs well wherever in ICU or OR and would not be influenced by low lung compliance, moderate PEEP and measurement devices. But TVC should be cautious applied in prone position and patients with spontaneous breathing activity.

## Supplementary Information

**Additional file 1: Fig. S1.** Graphs for meta-regression analysis. CI = confidence interval. Meta-regression was performed by position (supine or semi- recumbent vs. prone or Trendelenburg), PEEP ((5mH2O≤PEEP ≤15mH2O vs. others), Place (ICU vs. OR) and Device (TPTD vs. other measurement tools other than TPTD).

**Additional file 2: Table S1.** The influence of each trail for the meta-analysis.

**Additional file 3: Fig. S2.** Summary receiver operating characteristic curve for the change of pulse pressure variation after tidal volume challenge predicting fluid responsiveness in low tidal volume ventilation except Yonis 2017.

**Additional file 4: Fig. S3.** Sensitivity and specificity of the change of pulse pressure variation after tidal volume challenge predicting fluid responsiveness in low tidal volume ventilation for all data except Yonis 2017.

**Additional file 5: Fig. S4.** Summary receiver operating characteristic curve for the change of pulse pressure variation after tidal volume challenge predicting fluid responsiveness in ICU subgroup.

**Additional file 6: Fig. S5.** Summary receiver operating characteristic curve for the change of pulse pressure variation after tidal: volume challenge predicting fluid responsiveness in subgroups. a Supine or semi-recumbent group. b Lung compliance <30mH2O group. c Moderate PEEP group (5≤PEEP ≤15cmH2O). d Measurement tools without TPTD group.

**Additional file 7: Fig. S6.** Summary receiver operating characteristic curve for △PPV and △PPV% after tidal volume challenge predicting fluid responsiveness. A absolute change of pulse pressure variation (△PPV). B percentage change of pulse pressure variation (△PPV%).

## Data Availability

The datasets used and/or analyzed in the present study are available from the corresponding author on reasonable request.

## Abbreviations

PPV: Pulse pressure variation
△PPV: Absolute change of pulse pressure variation
△PPV%: Percentage change of pulse pressure variation
TVC: Tidal volume challenge
SROC: Summary receiver operating characteristic curve
AUSROC: Area under the summary receiving operating characteristic curve
AUROC: Area under the receiving operating characteristic curve
DOR: Diagnostic odds ratio
QUADAS-2: Quality assessment of diagnostic accuracy studies-2
TPTD: Transpulmonary thermodilution
PEEP: Positive end-expiratory pressure
CI: Cardiac index
SVI: Stoke volume index
SV: Stroke volume
ODM: Esophageal Doppler monitor
