# Supplementary figures and images for: Does tidal volume challenge improve the feasibility of pulse pressure variation in patients mechanically ventilated at low tidal volumes? A systematic review and meta-analysis
Source: Crit Care. 2023 Feb 2;27:45. doi: 10.1186/s13054-023-04336-6 (PMC9893685; doi:10.1186/s13054-023-04336-6)

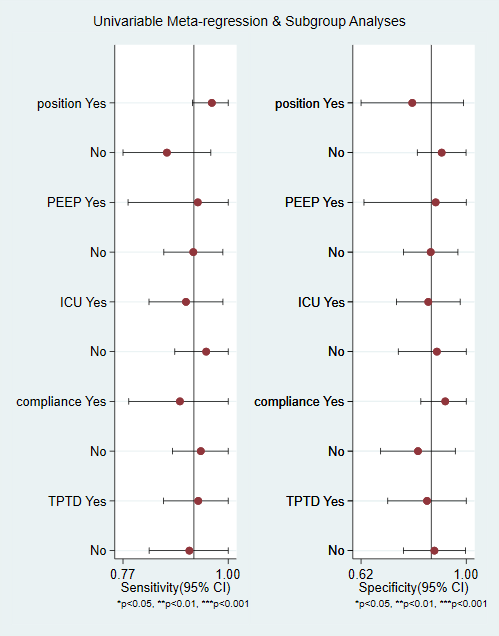

Supplement: Supplementary file 1 — Additional file 1: Fig. S1. Graphs for meta-regression analysis. CI = confidence interval. Meta-regression was performed by position (supine or semi- recumbent vs. prone or Trendelenburg), PEEP ((5mH2O≤PEEP ≤15mH2O vs. others), Place (ICU vs. OR) and Device (TPTD vs. other measurement tools other than TPTD). [file 13054_2023_4336_MOESM1_ESM.tif]

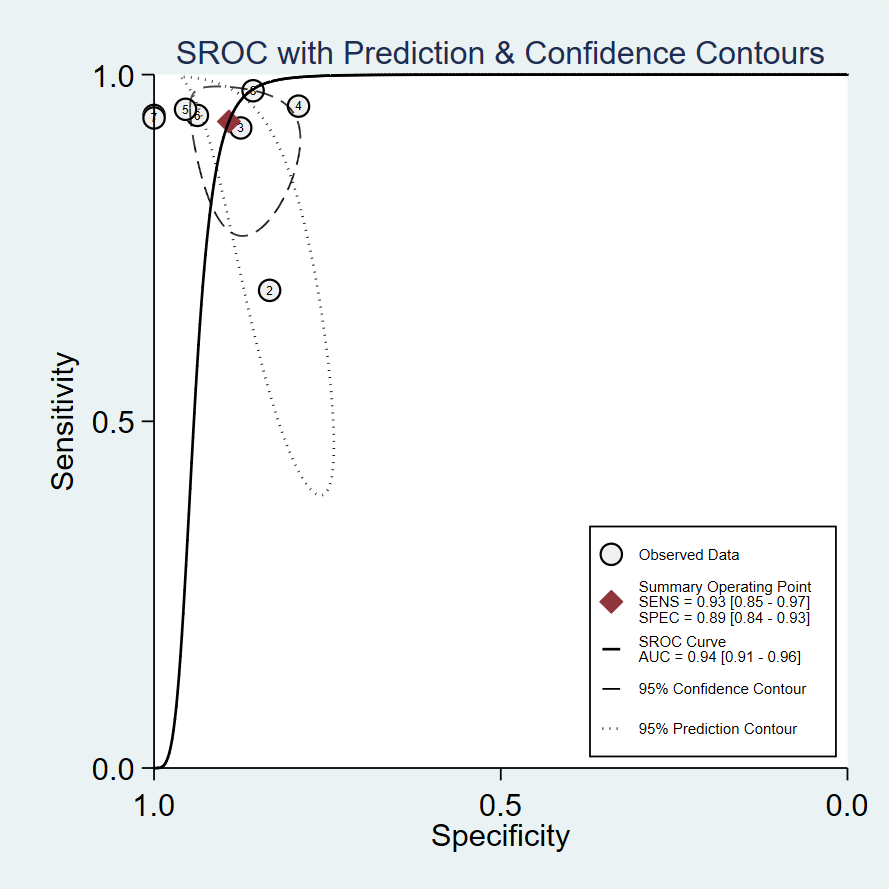

Supplement: Supplementary file 3 — Additional file 3: Fig. S2. Summary receiver operating characteristic curve for the change of pulse pressure variation after tidal volume challenge predicting fluid responsiveness in low tidal volume ventilation except Yonis 2017. [file 13054_2023_4336_MOESM3_ESM.tif]

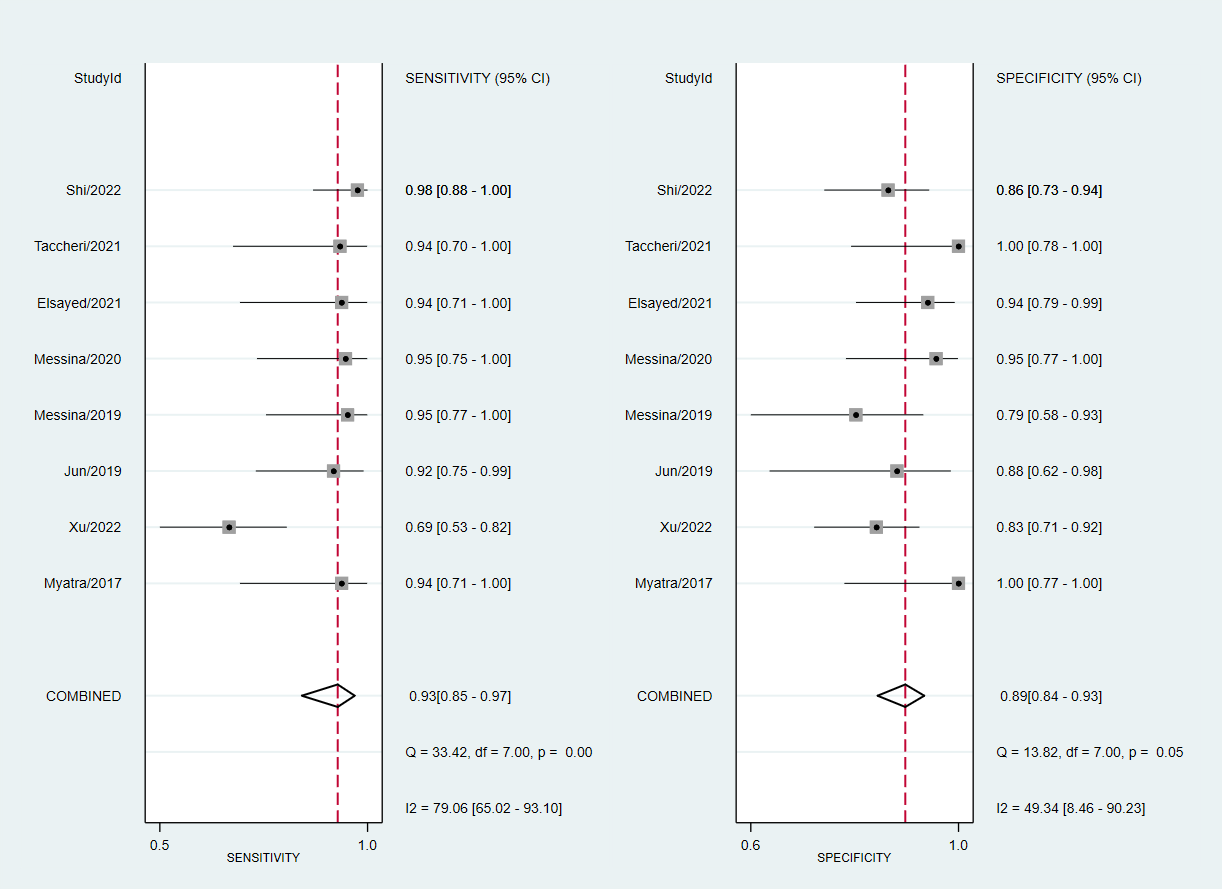

Supplement: Supplementary file 4 — Additional file 4: Fig. S3. Sensitivity and specificity of the change of pulse pressure variation after tidal volume challenge predicting fluid responsiveness in low tidal volume ventilation for all data except Yonis 2017. [file 13054_2023_4336_MOESM4_ESM.tif]

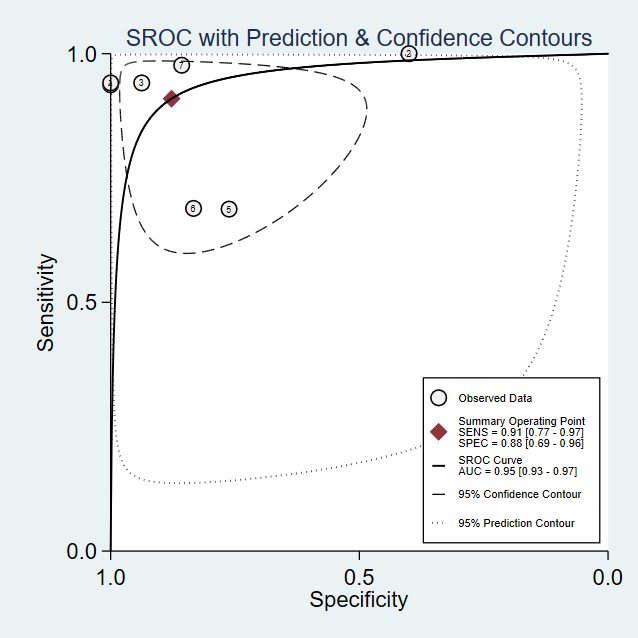

Supplement: Supplementary file 5 — Additional file 5: Fig. S4. Summary receiver operating characteristic curve for the change of pulse pressure variation after tidal volume challenge predicting fluid responsiveness in ICU subgroup. [file 13054_2023_4336_MOESM5_ESM.tif]

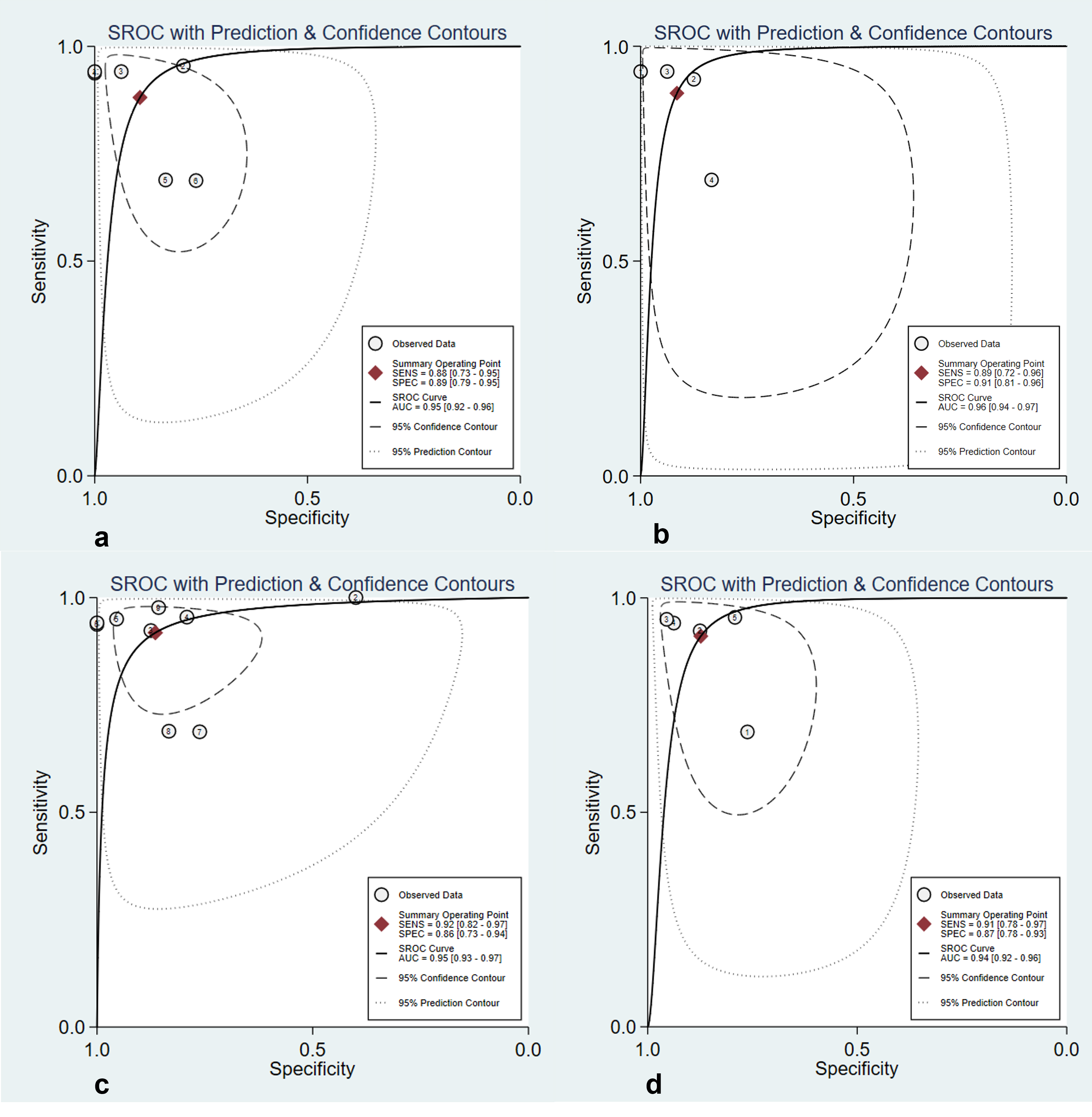

Supplement: Supplementary file 6 — Additional file 6: Fig. S5. Summary receiver operating characteristic curve for the change of pulse pressure variation after tidal: volume challenge predicting fluid responsiveness in subgroups. a Supine or semi-recumbent group. b Lung compliance <30mH2O group. c Moderate PEEP group (5≤PEEP ≤15cmH2O). d Measurement tools without TPTD group. [file 13054_2023_4336_MOESM6_ESM.tif]

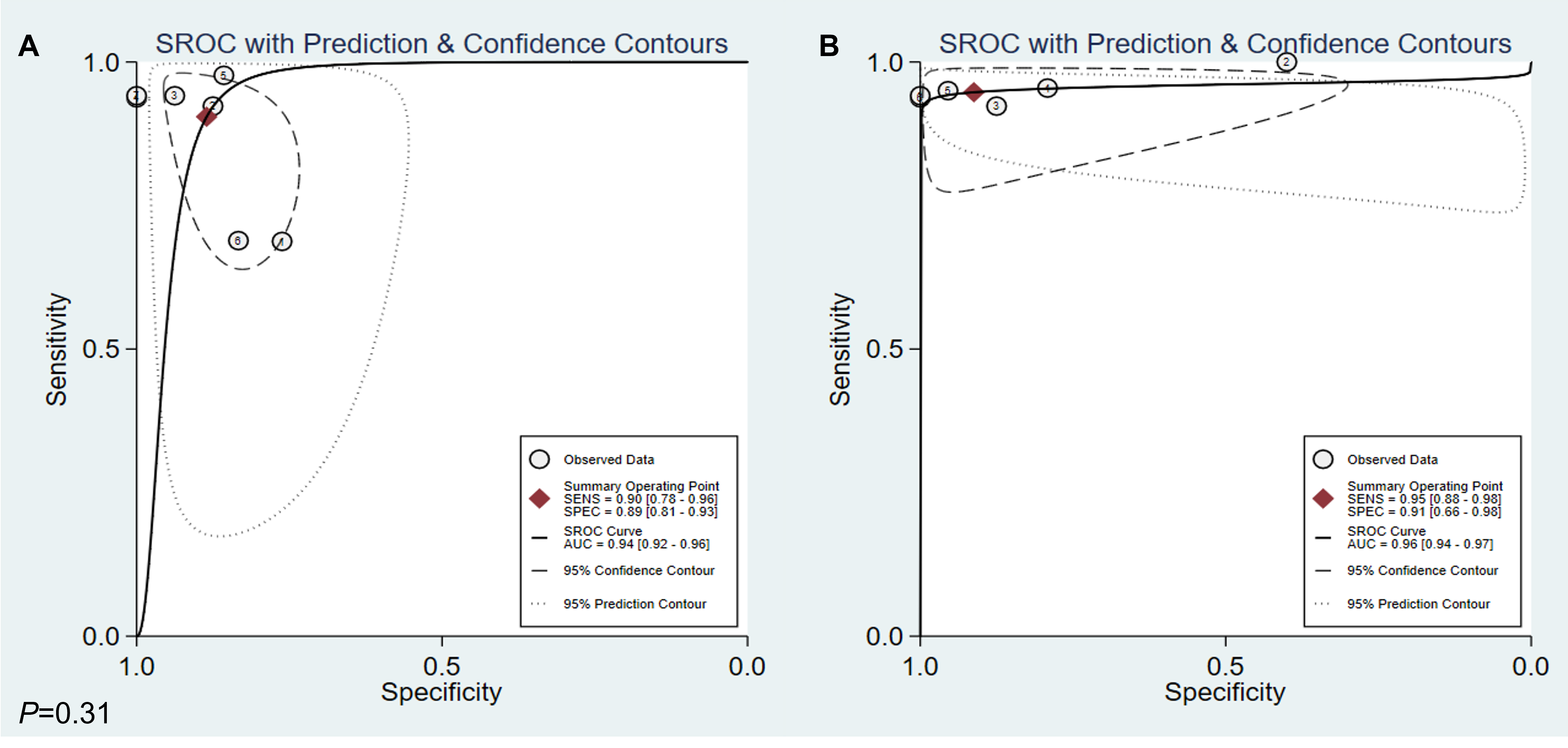

Supplement: Supplementary file 7 — Additional file 7: Fig. S6. Summary receiver operating characteristic curve for △PPV and △PPV% after tidal volume challenge predicting fluid responsiveness. A absolute change of pulse pressure variation (△PPV). B percentage change of pulse pressure variation (△PPV%). [file 13054_2023_4336_MOESM7_ESM.tif]
