# Supplementary material for: Does tidal volume challenge improve the feasibility of pulse pressure variation in patients mechanically ventilated at low tidal volumes? A systematic review and meta-analysis
Source: Crit Care. 2023 Feb 2;27:45. doi: 10.1186/s13054-023-04336-6 (PMC9893685; doi:10.1186/s13054-023-04336-6)
Supplement: Supplementary file 2 — Additional file 2: Table S1. The influence of each trail for the meta-analysis. [file 13054_2023_4336_MOESM2_ESM.docx]

**Table S1** The influence of each trail for the meta-analysis

| Except study | I^2^(%) | 95%CI | Q | *P* | DOR | 95%CI | AUROC | 95%CI | Spearman correlation coefficient | Proportion of heterogeneity likely due to threshold effect |
| --- | --- | --- | --- | --- | --- | --- | --- | --- | --- | --- |
| Myatra 2017[14] | 77 | (49,100) | 8.55 | <0.01 | 66 | (19,226) | 0.95 | (0.93-0.97) | 0.15 | 2% |
| Yonis 2017[15] | 42 | (0 , 100) | 3.5 | 0.09 | 83 | (26,260) | 0.94 | (0.92-0.96) | 1 | 100% |
| Jun 2019[16] | 78 | (51,100) | 8.96 | <0.01 | 84 | (21,341) | 0.96 | (0.94-0.97) | 0.22 | 5% |
| Messina2019[17] | 73 | (41,100) | 7.94 | 0.01 | 85 | (21,350) | 0.96 | (0.94-0.97) | 0.32 | 10% |
| Messina2020[18] | 77 | (50,100) | 8.78 | <0.01 | 68 | (19,245) | 0.95 | (0.93-0.97) | 0.16 | 3% |
| Elsayed2021[19] | 77 | (51,100) | 8.87 | <0.01 | 72 | (19,270) | 0.95 | (0.93-0.97) | 0.19 | 3% |
| Taccheri2021[20] | 77 | (49,100) | 8.51 | <0.01 | 66 | (19,226) | 0.95 | (0.93-0.97) | 0.16 | 3% |
| Hamzaoui2021[21] | 80 | (58,100) | 10.20 | <0.01 | 119 | (33,428) | 0.97 | (0.95-0.98) | -0.02 | 0% |
| Shi2022[22] | 70 | (34,100) | 6.75 | 0.02 | 72 | (17,292) | 0.95 | (0.93-0.97) | 0.35 | 13% |
| Xu2022[23] | 65 | (21,100) | 5.71 | 0.03 | 111 | (29,432) | 0.97 | (0.95-0.98) | 0.22 | 5% |

DOR, diagnostic odds ratio; CI, confidence interval; AUROC, area under the receiving operating characteristic curve；Q: Cochran’s Q test; I^2^: inconsistency

.
